# Presurgical localization of infected avascular bone segments in chronic complicated posttraumatic osteomyelitis in the lower extremity using dual-tracer PET/CT

**DOI:** 10.1186/s13550-018-0426-0

**Published:** 2018-07-21

**Authors:** Albert Christersson, Sune Larsson, Jens Sörensen

**Affiliations:** 1 Department of Orthopaedics, Institution of Surgical Sciences, Uppsala University, 751 85 Uppsala, Sweden; 2 Department of Nuclear medicine and PET, Institution of Surgical Sciences, Uppsala University, 751 85 Uppsala, Sweden

**Keywords:** Chronic osteomyelitis, FDG-PET/CT, NaF-PET/CT, Surgical treatment, Preoperative planning

## Abstract

**Background:**

Localizing and removing the infected sequestrum in long-standing trauma-related chronic osteomyelitis remains a clinical challenge. PET/CT with 18F-fluorodeoxyglucose (FDG-PET) has a high sensitivity for chronic osteomyelitis and 18F-sodium-fluoride PET/CT (NaF-PET) has a high specificity for identifying non-viable bone. Combining both, high signal on FDG-PET in the bone without signal on NaF-PET could potentially guide surgery to become more precise with curative intent.

Eight patients with long-standing (average 22 years) posttraumatic (*n* = 7) or postoperative (*n* = 1) chronic osteomyelitis in the lower extremity and with multiple futile attempts for curative surgery were recruited in this prospective pilot study. FDG-PET and NaF-PET were performed within a week in between using standard scanning protocols. The most likely location of the culprit sequestrum was identified and was surgically removed. Based on perioperative tissue cultures, antibiotics were given for 6–8 months. Dual-tracer (FDG- and NaF-PET/CT) was performed again after 12 months to rule out persisting signs of infection.

**Results:**

A likely culprit sequestrum could preoperatively be identified by dual-tracer PET in all eight cases and in four cases an additional sequestrum was identified at a location with no clinical sign of infection. The infected necrotic tissue was removed during surgery. Follow-up dual-tracer PET revealed no signs of persistent infection. All patients recovered with no clinical signs of recurrence for a follow-up of mean 4.5 (SD 1.3) years.

**Conclusions:**

Dual-tracer PET/CT with FDG and NaF allows successful precise surgery with curative intent in patients with long-standing complicated posttraumatic chronic osteomyelitis with severely deranged anatomy.

## Background

Infections following operative fixation of skeletal fractures, so-called fracture-related infections (FRI) [1] can be diagnosed by several imaging modalities. CT has low sensitivity and specificity but depicts bone structures, such as sequestrum, involucrum, and cloacae. MRI is able to identify involvement of the soft tissue, bone marrow, and joints, but postoperative scarring and oedema may result in false positive diagnosis, and artifacts from metal implants can prevent correct evaluation. MRI may also overestimate the extent of the infection due to its high sensitivity. Since CT and MRI mainly provide morphological information, they are often difficult to assess in peripheral skeleton when the anatomy is deranged after previous injury and therefore often combined with nuclear imaging modalities to get functional properties of the bone. Among these, white blood cell (WBC) scintigraphy or antigranulocyte antibody scintigraphy combined with SPECT/CT or 18F-fluorodeoxyglucose (FDG) PET/CT has the highest diagnostic accuracy [2–5]. WBC scintigraphy can also be used shortly after surgery with high accuracy [6]. PET is a technique that measures molecular properties of tissues using specific radiopharmaceuticals in tracer amount. 18F-fluorodeoxyglucose (FDG) PET/CT is commonly used for imaging in evaluation of malignancies, but is also very sensitive for osteomyelitis due to high accumulation of the tracer in activated leukocytes [7]. Both FDG-PET/CT and MRI have a high and comparable sensitivity for qualitative detection of chronic osteomyelitis. MRI is recommended in unifocal infections, but FDG-PET/CT is preferable in widespread or multifocal cases [8]. The specificity for FDG-PET/CT, as well as MRI, is low because it detects all kinds of inflammation involving metabolically active cells including anabolic processes during healing [7, 9, 10]. An important advantage for FDG-PET/CT compared with other nuclear imaging modalities is the improved spatial resolution, which makes it easier to localize increased uptake relative to the anatomy. In patients with long-standing posttraumatic chronic osteomyelitis (PTO), where the fracture already has healed and the metal implant has been removed, the clinical history with recurrent pain and fistulation is often sufficient for making the right diagnosis, and in these cases, the higher precision in PET/CT outweighs the problem with lower specificity.

One of the most notorious forms of FRI is a long-standing PTO, which is characterized by persistent infection in non-viable bone segments. PTO is difficult to cure, unless radical resection of necrotic bone can be achieved. Even though FDG-PET/CT detects the distribution of the osteomyelitis with high precision, this information is often not enough for a satisfactory preoperative planning. Assuming that the recurrent infection after systemic antibiotic therapy is caused by surviving bacteria in poorly perfused necrotic bone with low or no accumulation of antibiotics, the important issue preoperatively is not to localize the extension of the osteomyelitis in the bone but to localize the affected necrotic bone. CT, MRI, and FDG-PET/CT are all helpful but are limited regarding true location and distribution of the necrotic tissue. 18F-natrium-fluoride (NaF) is a highly bone-specific tracer, accumulating in the apatite in proportion to the rate of perfusion, mineralization, and bone formation [11]. NaF-PET/CT is increasingly used for visualizing the viability of the bone [12]. The higher precision of NaF-PET, compared to gamma-camera scintigraphy, is potentially helpful when planning a limited and precise resection of the bone, which does not threaten the integrity of the bone. The aim of this study was to examine the potential use of a dual-tracer PET/CT to localize the infected necrotic bone in patients with PTO in the lower extremity by first identifying the focal accumulation of activated inflammatory cells capable of migrating to the site inside the bone with FDG-PET/CT and then localizing the necrotic bone inside the inflammation with NaF-PET/CT. Our hypothesis was that a limited but precise surgical intervention based on a dual-tracer PET/CT could be both curative and result in low morbidity.

## Methods

This prospective case series consisted of eight male patients with a mean age of 52 (SD 11) years treated for chronic long bone osteomyelitis at the University Hospital of Uppsala, a tertiary referral center, between February 2010 and January 2016. Their history was basically similar, with chronic osteomyelitis after open high-energy fracture in the lower extremity in seven patients, and after ankle arthrodesis in one patient. The mean duration of chronic osteomyelitis was 22 (SD 13) years. All of the fractures were healed, and none of the patients had remaining osteosynthesis material when presented at our clinic. They had previously unsuccessfully been treated with one or more surgical revisions and antibiotics (Table 1).

**Table 1:** Characteristics of eight patients with posttraumatic (*n* = 7) or postoperative (*n* = 1) chronic osteomyelitis

Sex	Age (year)	Affected limb	Cause	Primary treatment	Duration (year)	Soft tissue	Sinus tract	Culture	Previous surgeries (*n*)^a^	Coverage with flap
Male	56	Femur dx	Traffic accident	Intramed nail, removed	37	Good	No	Staff Aureus, Propionibacterium	2	No
Male	28	Tibia sin	Bomb explosion	Conservative treatment	20	Poor	Yes, single	Escherichia coli, Klebsiella oxytoca	?	Yes
Male	57	Tibia sin	Traffic accident	External fixation	38	Poor	Yes, single	Staph Aureus	1	Yes
Male	53	Femur dx	Bomb explosion	Conservative treatment	22	Very poor	Yes, multiple	Proteus mirabilis Pseudomonas aeruginosa	4	Yes
Male	49	Tibia dx	Traffic accident	External fixation	12	Poor	Yes, single	Pseudomonas aeruginosa Propionibacterium	1	Yes
Male	58	Ankle arthrodesis	Rheumatoid arthritis	Screw fixation, removed	13	Poor	Yes, single	Pseudomonas aeruginosa	5	Yes
Male	55	Tibia dx	Traffic accident	Plate fixation, removed	2	Poor	Yes, single	Clostridium, Propionibacterium	1	Yes
Male	64	Femur sin	Traffic accident	Intramed nail, removed	33	Very poor	Yes, single	Serratia, Staph Epidermidis	7	Yes

^a^Number of documented failed previous surgical interventions intended to address the infection. Other surgical procedures are not included

The study was approved by the Ethical Committee of Uppsala University (Dnr 2015/146), and written informed consent was obtained from all patients according to the ethical guidelines of the Helsinki Declaration. All patients were preoperatively examined with FDG-PET/CT and NaF-PET/CT. All scans were performed with a GE Discovery ST16 hybrid PET/CT (Waukesha, ML) with routine clinical protocols. After at least 6 h fasting, FDG was injected intravenously 1 h before imaging (3 MBq/kg) and emission was acquired for 3 min per 15.7 cm bed position. NaF was injected at a dose of 2 MBq/kg 1 h before imaging with emission acquisition 2 min per bed position. CT without contrast enhancement was used for attenuation correction and was also reconstructed with a sharp bone-specific filter for visualization of anatomy. PET images were reconstructed with all relevant corrections using standard clinical protocols. FDG-PET and NaF-PET were performed at two different days to avoid interference between the tracers, and the images were fused with the simultaneously acquired CT for precise anatomic orientation of the uptake. The uptake on FDG-PET and NaF-PET were carefully compared to each other and a demarcated high uptake on FDG-PET in an area with low or absent uptake on NaF-PET inside the bone, as seen on CT, indicated inflammation in necrotic bone and was considered to be a sequestrum harboring infection. Standard uptake values (SUV) were recorded as max SUV in the FDG culprit lesion, as max SUV in nearest viable bone with reactive NaF uptake, and as SUV mean in a corresponding circular region with a 2-cm diameter in the contralateral leg.

Five (5/8) patients with signs of active infection (redness and swelling) were treated with adequate antibiotics for at least 4 weeks prior to and throughout the PET examinations based on previous cultures to reduce the area on FDG-PET affected by the infection, assuming that imaging under ongoing antibiotic treatment would minimize the area of infection for easier identification of the infected sequestrum. The remaining three (3/8) patients had no signs of acute infection and were therefore not treated with antibiotics prior to or during the PET examinations. All antibiotics were withdrawn 4 weeks preoperatively to make cultures more reliable. For localizing the potential sequestrum, the images from FDG-PET and NaF-PET were examined simultaneously in a parallel manner in axial, coronal, and sagittal sections. Following a thorough preoperative planning, all patients were operated with resection of the presumed necrotic bone. The skin incision in seven (7/8) patients included excision of a sinus tract and the surrounding affected skin, thereby facilitating the access to the bone. The preoperatively identified bone fragments were excised, and the bone cavity was curettaged and thoroughly irrigated. Multiple tissue cultures were taken for confirmation of the diagnosis. Local antibiotic (calcium sulphate (Rapid Cure®) with Vancomycin) came onto the market in 2014 and was used in the last two patients. The integrity of the bone was preserved in all cases and no stabilization with osteosynthesis was needed. All but one patient needed a concomitant coverage with a soft tissue flap. The patients were allowed to full weight bear after the operation. Postoperatively, the patients were given antibiotics intravenously for 1 week, and based on the perioperative tissue cultures (Table 1), they were given antibiotics orally for 6–8 months. FDG-PET/CT and NaF-PET/CT were repeated 12 months after the operation, i.e., at least 4 months after withdrawal of antibiotics. Absence of uptake on FDG-PET at 12 months and absence of wounds and local symptoms in the extremity was considered to be a curative successful treatment.

## Results

**Fig. 1:**
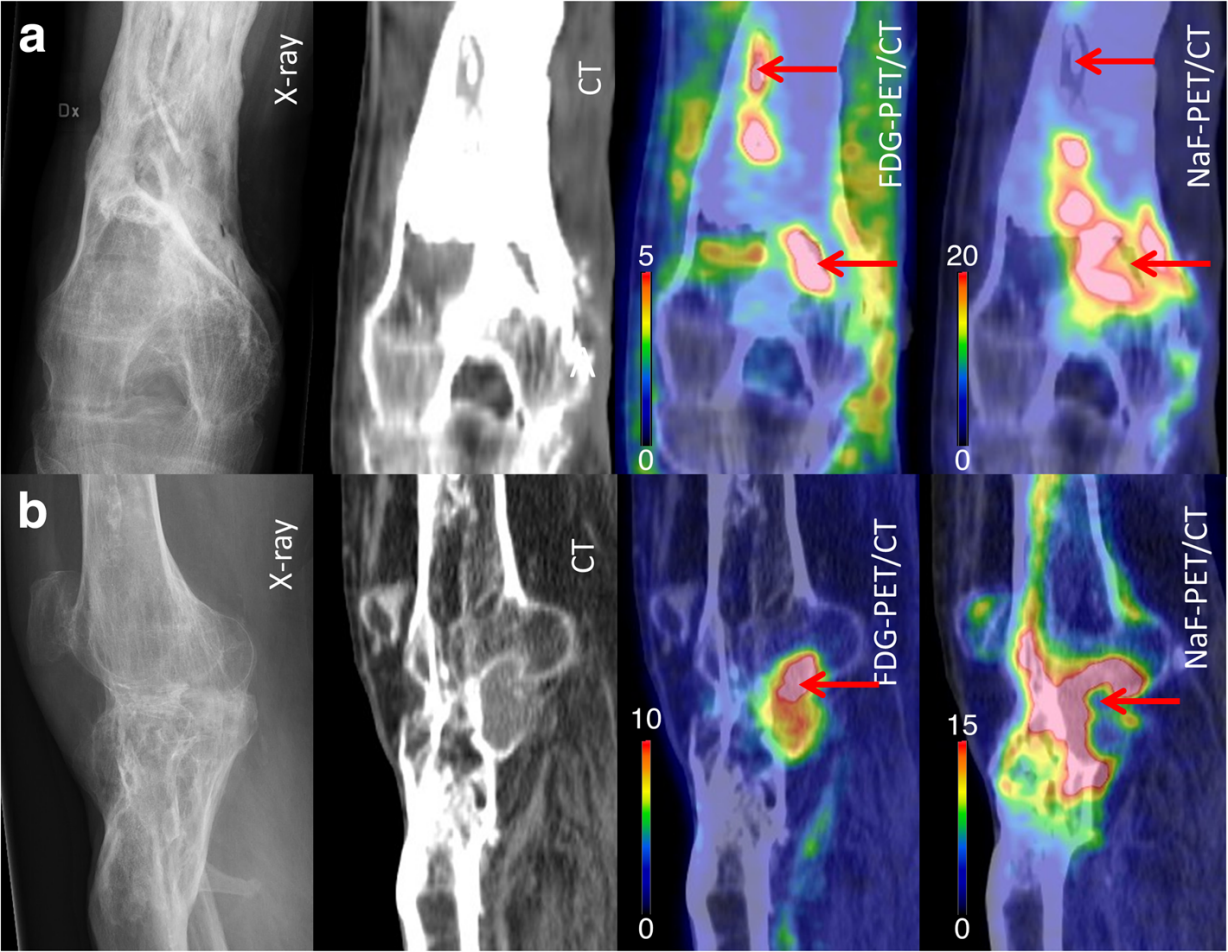
Preoperative images (X-ray, CT, FDG-PET/CT, and NaF-PET/CT) of chronic osteomyelitis in distal femur in two different patients. The arrows point at bone segments with high uptake on FDG-PET/CT (“hot spots”) and low uptake on NaF-PET/CT (“cold spots”). The findings are best visualized in coronal views in patient (**a**), where two sequestra were found, and in sagital views in patient (**b**), where one sequestrum was found

In all eight (8/8) preoperative dual-tracer PET/CT, at least one small intraosseous area with high uptake on FDG-PET and absent uptake on NaF-PET was localized, which were assumed to be the sequestrum (Fig. 1). Median FDG SUVmax was 5.8 g/ml (range 2.4–11.4) and the ratio of hotspot/contralateral region 14.4 (range 3.1–28.5). In four patients, a secondary area with an additional sequestrum was found, separated from the major infection, and located where we primarily had no suspicion of infection. Typically, NaF uptake was highly elevated in the bone adjacent to the sequestrum. Median NaF SUVmax was 14.5 g/ml (range 2.6–47.9) and hotspot/contralateral ratio 12.9 (range 3.3–43.6). The lowest NaF SUV (“coldspot”) at the site of the FDG hotspot was median 5.2 (range 2.2–11.8), which was 4.1 (range 1.7–9.6) times higher than contralateral uptake, but also 3.5 (range 3.1–4.9) times lower than the NaF hotspot. SUVmax from FDG and NaF hotspots correlated (Spearman rho = 0.82, *p* < 0.05). Perioperative tissue cultures were positive in all eight patients, confirming the diagnosis of PTO. Twelve months after the operation, seven (7/8) patients had complete disappearance of the preoperatively detected increase in FDG-PET uptake. The remaining patient had on the preoperative dual-tracer PET/CT, besides the most obvious area of necrotic tissue inside the bone, an additional small area of increased FDG-PET uptake localized in the soft tissue a few centimeters away from the bone. This area had no uptake on the NaF-PET. This finding was preoperatively overlooked, and it was still present on the postoperative dual-tracer PET/CT 12 months postoperatively. When this area was surgically explored, we found a granuloma with a piece of encapsulated textile from the primary open injury. All eight patients recovered clinically after surgery and are still free from wounds and antibiotics at the time of writing (4.5 (SD 1.3) years after surgery).

## Discussion

This is, to the best of our knowledge, the first paper to describe the combined use of FDG- and NaF-PET in the preoperative planning of long-standing chronic osteomyelitis after trauma. Preoperative FDG-PET in patients with PTO without clinical signs of present acute infection (in 5/8 patients achieved by on-going antibiotic therapy) localized the distribution of the chronic infection as “hot spots.” In a few cases, FDG-PET disclosed increased uptake in adjacent bone parts initially not suspected to be affected, such as extensions in direct contact with the major infection but also detached areas away from the major infection. NaF-PET localized necrotic bone as “cold spots,” which, in combination with high uptake on FDG-PET, revealed the precise location of the sequestra. A meticulous preoperative planning made it possible to get an adequate access to the infections. The operations with free soft tissue flaps in seven (7/8) of the patients contributed to the successful treatment of the infections, but since chronic osteomyelitis cannot be cured without resection of all infected necrotic bone, cure had not been possible with soft tissue coverage alone.

The two tracers used represent distinctly different biological entities. FDG is taken up by a multitude of different cell types involved in inflammation, infection, and bone remodeling, including not only activated leucocytes but also activated fibroblasts. FDG uptake is typically found in healing fractures and bone undergoing remodeling, potentially inducing false positive findings in the setting of localizing infection. NaF, on the other hand, is specifically accumulated in the matrix surrounding activated osteoblasts and free mineral adjacent to perfused tissue [13]. The uptake of NaF is always elevated in healing bone and anabolic bone remodeling.

The high uptake of FDG in poorly perfused necrotic bone is remarkable. This seems to indicate a constant and rapid migration of leucocytes and other inflammatory cells into the epicenter of the infection leading to an accumulation of FDG in or around the sequestrum. Neutrophils move at a speed of approximately 20 μm/min when exposed to specific chemoattractants [14]. Theoretically, white blood cells containing FDG can migrate a maximum of 1.2 mm during the 1 h time span from injection of FDG to scanning. The absent or very low uptake of NaF in the necrotic bone suggests that the diffusion distance of fluoride ions is reduced or that binding to the mineral is repelled by a biological mechanism, i.e., bacterial biofilm [15]. The hypoperfused core of a necrotic bony sequestrum harboring bacteria is therefore less accessible for fluoride ions, resulting in a “cold spot” in the NaF-PET images. This is probably also the rationale for the poor penetrance of antibiotics into the core of the sequestrum. Localizing a cold spot on NaF-PET in an area of a “hot spot” on FDG-PET was essential for making a limited but still precise resection of the bone tissue, not endangering the integrity of the bone. As shown here, NaF uptake adjacent to the FDG hot spot can be substantial and NaF uptake was never zero at the epicenter of the FDG hot spot. If the sequestrum is small there will be “spill-in” of activity into the sequestral region, but a relative NaF cold spot could always be seen.

A preoperative planning based on FDG-PET alone had resulted in more extensive bone exposure and resection since perioperative assessment of bone viability is a recognized difficult issue. The pursuit of being radical in excising the necrotic bone, and perioperatively only relying on the viability of the bone (i.e., signs of bleeding), increases the risk for removing too much bone, potentially leading to bone instability and a need for fracture fixation, which results in postoperative morbidity. Alternatively, the infection recurs if not all necrotic bone is extirpated. The addition of NaF-PET to FDG-PET seems to improve the accuracy, and we found it essential for a successful outcome in seven (7/8) operations. The dual-tracer PET was very helpful to localize and demarcate the sequestrum in these patients. In addition, in four (4/8) of these cases, the dual-tracer PET localized an additional sequestrum. This information was crucial for curative surgical treatment. In one (1/8) patient, with a small unifocal osteomyelitis, the extra information provided by the NaF-PET did not change the surgical procedure, and in this patient, a good result had probably been achieved with FDG-PET alone.

The interpretation of the dual PET is time-consuming and has a learning curve, which makes it user-dependent. A good postoperative outcome relies on a thorough preoperative planning including positioning of the patient, surgical approach, and location for entrance into the bone based on the dual-tracer PET/CT.

A limitation of the study is the short follow-up time. A chronic osteomyelitis can remain inside the bone for many years without any clinical symptoms and recur later. A negative FDG-PET after 12 months, and the absence of clinical symptoms during a few subsequent years, increases the probability of cure, but leaves no absolute guarantee [16].

It is also a limitation that the study has no control group, and no comparison to more commonly used imaging techniques. It is possible that the same good result could have been achieved after preoperative evaluation with CT, MRI, or hybrid imaging nuclear modalities like WBC scintigraphy or antigranulocyte antibody scintigraphy with SPECT/CT. Early dynamic 18F-NaF potentially has the ability to both differentiate infected tissue from healthy tissue and localize necrotic bone [17]. Indeed, if a bone, affected with unifocal PTO, does not have a severely deranged anatomy, CT, or MRI can be sufficient for diagnosis and preoperative planning. It is our experience that dual-tracer PET/CT might have its greatest potential in complicated longstanding posttraumatic cases with severely deranged anatomy when CT and MRI are unable to demonstrate the number and true localization of sequestra.

## Conclusions

Dual-tracer PET/CT with FDG and NaF provides information about the location of the infected sequestra in patients with complicated long-standing posttraumatic chronic osteomyelitis with severely deranged anatomy, which allows successful precise surgery with curative intent.
